# Key quality factors for Chinese herbal medicines entering the EU market

**DOI:** 10.1186/s13020-022-00583-x

**Published:** 2022-02-22

**Authors:** Mei Wang, Pei-Fen Yao, Peng-Yue Sun, Wen Liang, Xiao-Jia Chen

**Affiliations:** 1 LU-European Center for Chinese Medicine and Natural Compounds, Institute of Biology, Leiden University, Sylviusweg72, 2333BE Leiden, The Netherlands; 2 Institute of Chinese Medical Sciences, and State Key Laboratory of Quality Research in Chinese Medicine, University of Macau, Avenida da Universidade, Taipa, Macao SAR People’s Republic of China; 3 SU Biomedicine, BioPartner Center 3, Galileiweg 8, Leiden Bio Science Park, 2333 BD Leiden, the Netherlands; 4 Zhuhai UM Science & Technology Research Institute, Zhuhai, 519031 China

**Keywords:** Chinese herbal medicines, European Pharmacopoeia, Chinese Pharmacopoeia, Quality requirements, European herbal medicine legislation, European market

## Abstract

Chinese herbal medicines (CHMs) have unique advantages in the prevention and treatment of diseases, which are widely recognized in the world. More and more CHMs are becoming increasingly popular in the international markets. However, the quality control of CHMs is a significant issue for their acceptance and recognition in the international market. This review mainly focuses on the quality requirements for CHMs to enter the European Union (EU) market. Both Chinese and European regulations and quality controls are compared. Firstly, the EU medicinal regulatory system and relevant regulations were reviewed. Secondly, the key factors of the quality control of CHMs, including Chinese herbal drugs, extracts and products were compared with those of European herbal medicines in the EU market. Subsequently, three main registration routes for herbal medicinal products including Chinese herbal medicinal products entering the EU were introduced. Furthermore, the legal status of traditional Chinese medicine granules in the EU was also discussed. Through the comparison of the key quality factors for CHMs in China and the EU, the similarities and differences in terms of quality requirements and regulations are addressed, which provides a reference for the development of CHMs into the EU market.

**Supplementary Information:**

The online version contains supplementary material available at 10.1186/s13020-022-00583-x.

## Introduction

Chinese herbal medicines (CHMs) are defined as Chinese herbal drugs, Chinese herbal drug extracts, Chinese herbal medicinal products and traditional Chinese medicine granules (TCM granules) in this review. The use of CHMs should be under the guidance of traditional Chinese medicine (TCM) theories. TCM theories consider that CHMs have *four properties*, including *heat, warm, cold and cool*, and *five flavors*, including *sweet, acrid, salty, sour and bitter* [1]. For improving therapeutic effects and reducing toxicity, many Chinese herbal drugs need to be processed before clinical applications [2]. Thus, several traditional processing methods have been developed, including cutting, crushing, steaming, calcining, and stir-frying with or without liquid/solid excipients (honey, Chinese yellow wine, Chinese vinegar, salt, soil, rice wheat bran etc.) [2–4]. The classic dosage forms of traditional Chinese herbal medicinal products include decoction, pill, powder, concentrated decoction, ointment, plaster, syrup, wine, suppository, tincture and so on [5]. With the development of modern sciences and technologies, the dosage forms have been greatly enriched, encompassing tablets, capsules, granules, dripping pills, inhalants and so on [5, 6]. The personalized interventions and the holistic view are two important characteristics for treatments with CHMs, taking advantage of multi-targets and multi-components intervention strategies [7]. A significant amount of historical records, case studies and results of evidence-based clinical trials demonstrate CHMs play an important role in the treatment and prevention of diseases such as cardiovascular diseases [8], liver damages [9], cancers [10], acute respiratory distress syndrome [11] and even recently COVID-19 [12, 13]. Therefore, the values of CHMs in the field of healthcare are widely recognized [14] and more and more people worldwide have used them in daily life for various health-related issues [15].

With the worldwide application of TCM, CHMs have entered the international market quickly. Meanwhile, their scientific evidence, safety issues/possible toxicities and quality issues have also gained a lot of attention [16]. For protection of consumers’ health benefits, several countries and regions have started to establish quality standards for CHMs in their national pharmacopoeias and regional standards [17]. In Europe, since 2009, Chinese herbal drug quality monographs have been gradually established and elaborated by the TCM working party in the European pharmacopoeia (Ph. Eur.) [4].

A pharmacopoeia is a book containing directions for the identification of compound medicines and published by the authority of a government or a concerned pharmaceutical society. Descriptions of preparations are called monographs. It is a crucial reference work for all organizations and individuals in the field of pharmaceutical research and development, manufacture and testing around the world [17]. The mainly representative national pharmacopoeias include the Chinese Pharmacopoeia (ChP), the Ph. Eur., the Japanese Pharmacopoeia (JP), and the United States Pharmacopeia (USP)-National Formula (NF). In addition, there are some regional and/or commercial standards with herbal monographs, e.g., the Hong Kong Chinese Material Medica Standards, the American Herbal Pharmacopoeia, the traditional Chinese Medicine standards of the International Organization of Standardization, and herbal monographs of the World Health Organization [17–19]. In different pharmacopoeias, there is a huge variation in the numbers of recorded Chinese materia medica. For example, the 2020 edition of the ChP records 2711 monographs of Chinese materia medica including herbal drug, animal-sourced materia medica and mineral-sourced materia medica [20]; however, there are relatively fewer herbal drug and/or herbal extract monographs in the Ph. Eur., the JP and the USP-NF. No animal-sourced materia medica and mineral-sourced materia medica are elaborated in the Ph. Eur. In addition, since the quality control systems of herbal drugs are different in each country or region, monographs of CHMs could be different in different pharmacopoeias.

Data provided by the Chinese Chamber of Commerce for Import and Export of Medicine and Health Products showed that the import and export values of CHM products to the European Union (EU) reached approximately 850 million USD in 2017, suggesting a high market share of CHMs in the EU [21]. Furthermore, there is still a huge market capacity available, compared with the 5.19 billion USD of total trade volumes in 2017 [21]. For the acceptance of CHMs into the EU market, the requirements of CHMs and the medicinal regulatory system and relevant regulations in the EU need to be understood and reviewed systematically. This review mainly focuses on the key factors of quality evaluation of CHMs and explores the pathways for CHMs to enter the EU market via in-depth interpretation of the relevant requirements of laws and regulations in the EU. Meanwhile, in order to help Chinese research organizations/pharmaceutical companies to understand the difference in laws and regulations between the EU and China, the comparative discussion is included in this review.

## Brief description of the EU medicinal regulatory system

**Fig. 1:**
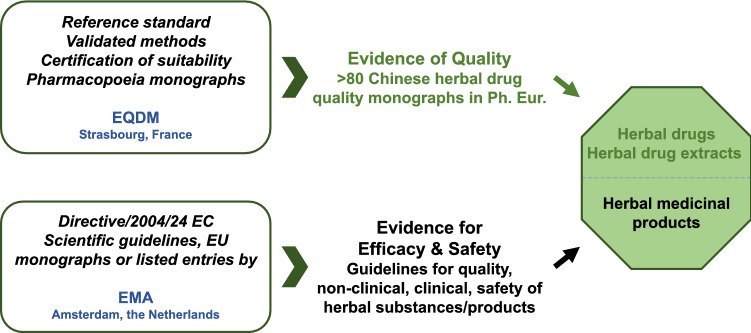
Different roles of the EDQM and the EMA for regulation of herbal medicinal products in the EU. Within the EU, the Ph. Eur. under management of the EQDM is a legally binding document for quality standards for medicinal products. The EMA is mainly responsible for efficacy and safety of medicinal products. The combination of both institutions plays central roles in medicinal products. The different terms are used: Herbal drug (Ph. Eur.) = herbal substance (EMA); herbal medicinal product (Ph. Eur. and EMA)

The European medicinal regulatory system is based on a network of the regulatory authorities from the European Economic Area countries: the European Commission, the European Medicines Agency (EMA) and the European Directorate for the Quality of Medicines and Health Care (EDQM), making the EU regulatory system unique in the world. In this system, the EMA and the EDQM play core roles in the medicinal registrations and quality managements (Fig. 1).

The EMA is a decentralized agency and is responsible for the establishment and explanation of scientific guidelines and the EU monographs, drafting an EU list of herbal substances, preparations, and combinations for use in traditional herbal medicinal products, and the scientific evaluation, supervision and safety monitoring of medicines, etc. on behalf of the European Commission. To be noticed worldwide, the term “EU monographs” provides scientific evidence for safety and efficacy for herbal medicinal products, which is different from the monograph of the Ph. Eur. that provides scientific evidence for the quality of herbal drugs [22, 23]. In the EU, medicinal products including herbal medicinal products are authorized/registered by the European Commission/EMA or the medicine agencies at national level in the EU. The EDQM is an organization of the Council of Europe that protects public health by enabling the development, supporting the implementation, and monitoring the application of quality standards for medicines and their safe use.

The EDQM develops the Ph. Eur., which covers the quality requirements for a wide range of substances, including herbal drugs and extracts. Meanwhile, general quality matters are also addressed in scientific guidelines issued by the EMA such as the declaration of herbal substances (in the Ph. Eur., also named herbal drugs) and herbal preparations in herbal medicinal products, quality of herbal medicinal products, specifications: test procedures and acceptance criteria for herbal substances, herbal preparations and herbal medicinal products. The EMA and the EDQM play in concert for the regulation of herbal drugs/herbal extracts and herbal medicinal products in the market. Therefore, herbal drugs or herbal extracts should at least meet these quality standards in the Ph. Eur. monographs, when applying for marketing authorization (MA) or registration of their medicinal products in the EU (Fig. 1).

The EU pharmaceutical legislation makes direct reference to the Ph. Eur. and other quality related issues which are under the EDQM responsibilities, such as certification of suitability to the monographs of the Ph. Eur. and co-ordination of the European network of Official Medicines Control Laboratories. The EMA and national competent authorities are responsible for evaluation of the safety and efficiency of medicinal products. This demonstrates there is close cooperation between these European organizations in the protection of public health (Fig. 1). The requirements and procedures for MA/registration, as well as the rules for monitoring authorized/registered medicinal products, are the tasks of the EMA and primarily laid down in Directive 2001/83/EC and in Regulation (EC) No 726/2004 which are legally binding documents. They also include harmonized provisions for the manufacture, wholesale, or advertising of medicinal products for human use.

## Comparison of CHMs and European herbal medicines in quality requirements

### Comparison of the quality requirements for Chinese and European herbal drugs

Herbal drugs are the starting materials in manufacturing chain of herbal medicinal products, including Chinese herbal medicinal products. The quality assurance of herbal drugs is essential to guarantee the quality of their extracts and finished products. So, quality is the key point when CHMs enter the EU market. Generally, the quality requirements of herbal drugs in the EU are mainly defined in the Ph. Eur., which is similar in China, namely the quality standards of Chinese herbal drugs are mainly defined in the ChP.

For the European herbal drugs, the quality monographs have been established by a group of scientists/experts named working party 13A and 13B at the EDQM, which consists of the scientists/experts from different member states. For the Chinese herbal drugs in the Ph. Eur., the quality monographs have been set up by a TCM working party, which consists of the scientists/experts from both different member states and China. The TCM working party was established by European Pharmacopoeia Commission at the EDQM in 2008 [4]. Until now, there have been over 80 Chinese herbal drugs recorded in the Ph. Eur. and more will surely follow [22].

In the Ph. Eur., a general principle monograph on quality standards for herbal drugs is established to outline the quality monograph of an herbal drug, which contains universal tests and acceptance criteria, such as identification, tests for foreign matter (*2.8.2*), loss on drying (*2.2.32*), water (*2.2.13*), pesticides (*2.8.13*), heavy metals (*2.4.27*), total ash (*2.4.16*), ash insoluble in hydrochloric acid (*2.8.1*), extractable matter, swelling index (*2.8.4*), bitterness value (*2.8.15*), aflatoxin B, (*2.8.18*), ochratoxin A (*2.8.22*), radioactive contamination, and microbial contamination (*5.1.8.* or *5.1.4.*) [24]. The special analytical procedures and acceptance criteria for the identification, test, and assay of each herbal drug are described in the quality monograph of individual herbal drugs. In the ChP, there is not only a similar general principle of monograph on quality standard for Chinese herbal drugs, but also a unique general principle of monograph for processed Chinese herbal drugs in Volume IV of the ChP. In both pharmacopoeias, the key points of quality control could be found in both general principle of monograph and the quality monograph of individual herbal drugs.

The essential contents of an herbal drug monograph in the Ph. Eur. are quite like those in the ChP, which contain the definition of an herbal drug, as well as its identification, test, assay, etc. Both pharmacopoeias also apply similar concepts for quality control or analytical methods such as macroscopic and microscopic description and thin layer chromatography (TLC) for identification, foreign matter, loss on drying, total ash, etc. for tests, liquid chromatography for assay, although there are still many variations in detailed requirements [22].

Meanwhile, there are some significant differences in expression between them, e.g., TLC is usually expressed as a characteristic fingerprint after treatment in the Ph. Eur., while it is normally described in words in the ChP, e.g., by comparison with spots of the reference herbal drug and/or the chemical reference substances in the TLC chromatogram, same color or fluorescent spots of the test solution are displayed on the corresponding positions of the chromatogram. In addition, the other main differences between them involve some specific technical requirements such as pesticide residues, heavy metals, and aflatoxins. For pesticide residue, there are total of 69 pesticide residue limits recorded in the Ph. Eur. (Additional file 1: Table S1), which involve the limits of organochlorine pesticides, organophosphorus pesticides, pyrethroid pesticides, etc., while 33 pesticide residue limits are recorded in the ChP (Additional file 1: Table S2). For heavy metals, the general limits for herbal drugs in the Ph. Eur. are as follows: cadmium (≤ 1.0 ppm), lead (≤ 5.0 ppm), and mercury (≤ 0.1 ppm), but special requirements may be needed for some herbal drugs such as Lini Semen (cadmium ≤ 0.5 ppm), and there are no requirements for arsenic and copper. In the ChP, there is a guiding standard for heavy metals, which are cadmium (≤ 1 ppm), lead (≤ 5 ppm), mercury (≤ 0.2 ppm), arsenic (≤ 2 ppm) and copper (≤ 20 ppm), respectively, and 20 Chinese herbal drugs are required for heavy metal test compulsorily (Additional file 1: Table S3). For aflatoxins, in the Ph. Eur., all herbal drugs are required the detection of aflatoxins. In the ChP, the limitation of aflatoxins is gradually being improved. The 2005 edition of the the ChP Supplement contains the "Aflatoxin Assay" in appendix for the first time. Subsequently, the 2010 edition of the ChP increased aflatoxin screening targets and limitation standards for 4 Chinese herbal drugs, including Ziziphi spinosae semen, Persicae semen, Sterculiae lychnophorae semen and Citri reticulatae pericarpium [25]. The number of Chinese herbal drugs which are required to test aflatoxins increased to 14 in 2015 edition of the ChP. While in 2020 edition of the ChP, there are 16 Chinese herbal drugs to be required the limitation of aflatoxins, and the limits are aflatoxin B_1_ (≤ 5 μg/kg) and sum of aflatoxin G_2_, aflatoxin G_1_, aflatoxin B_2_, aflatoxin B_1_ (≤ 10 μg/kg) (Additional file 1: Table S4). In addition to aflatoxins, in the 2020 edition of the ChP, Coicis semen is also required to test its zearalenone (≤ 500 μg/kg) [23].

### Comparison of the quality requirements for Chinese and European herbal drug extracts

Both the Ph. Eur. and the ChP contain monographs of herbal drug extracts obtained from herbal drugs using suitable solvents, whose physical form could be liquid (liquid extraction preparations), semi-solid (soft extracts and oleoresins) or solid (dry extracts) preparations, respectively. In the Ph. Eur., extracts can be principally classified to be either a standardized extract, quantified extract, or other extract, respectively. This classification is based on clinical validation of chemical composition, or determination of marker compounds and others, etc. (Table 1).

**Table 1:** Herbal extracts in the Ph. Eur

Items	Classifications	Characteristics
Based on chemical constituents/markers	Standardized extracts	1. Chemical constituents/markers with known therapeutic activity 2. A defined content, single content or single content in a defined range, of one or more the active chemical constituents/active markers, with acceptable tolerance within the range ± 5% to ± 10% taking into account the nature of the extract and the method of assay 3. Blending batches of the extract, or adjustment with inert excipients, is available
	Quantified extracts	1. A limited and specified range of content with one or more active markers 2. Blending batches of the extract is available
	Other extracts	The minimum content for one or more analytical markers is required
Based on physical states	Liquid extraction preparation/liquid extracts	1. Standardized liquid extracts 2. Quantified liquid extracts 3. Other liquid extracts
	Liquid extraction preparation/tinctures	1. Standardized tincture 2. Quantified tinctures 3. Other tinctures
	Soft extracts	Semi-solid preparations obtained by evaporation or partial evaporation of the solvent used for production
	Oleoresines	Semi-solid extracts composed of a resin in solution in an essential and or fatty oil
	Dry extracts	Solid preparations by evaporation of the solvent with a loss on drying of not greater than 5% *m/m,* where justified and authorized, with a different limit available

Some of technical terms are seldom used or not used at all in the CHMs industry such as drug extract ratio (DER) and drug extract ratio _genuine_ (DER _genuine_), which are briefly described as follows. In Europe, DER or DER _genuine_ is an important concept and is widely used to define extracts in herbal medicinal products. DER is the ratio of the quantity of herbal drug used in the manufacture of an extract and the quantity of extract obtained, and DER _genuine_ refers to the ratio of the herbal drug and the extract without excipients. Both will be identical without processing aids, otherwise, they have different values. As a technical term, percentage of herbal extracts, or extraction rate, or extraction yield, is often used in technical documents for CHMs in China. But, when placing CHMs in the EU market, the concept of DER or DER _genuine_ needs to be used in labeling, summary of product characteristics, EU herbal monograph, etc. Among these two terms, their basic logics are the same although their expressions are different [26].

Regarding the purification of an extract, the degree of purity designated as an herbal preparation needs to be considered carefully. It can lead to problems sometimes when preparing a dossier for registration in the EU. This is a frequent issue in the registration of a Chinese herbal medicinal product in the EU. The level of refinement is actually an important factor in defining an herbal extract, therefore, a careful evaluation of the borderline is needed to ensure that the correct category is assigned to establish the appropriate specifications and regulatory status. It is pity that there is no such requirement in either the Ph. Eur. or the ChP. However, there is a discussion about this in the EMA. When assessing herbal extracts in this case, at least, one of the following criteria would exclude the material being designated as a ‘herbal preparation’: (1) Extracts subjected to chemical processes where the chemical modifications may be comparable to a partial synthesis. These preparations should be assessed case by case. (2) Extracts enriched with isolated compounds. The following aspects should be considered in designating the extract as a ‘herbal preparation’: (1) Definition is in accordance with Directive 2001/83/EC as amended and the Ph. Eur. (2) A complex mixture of constituents extracted from plant material is present in the preparation. (3) The proportional content of constituents in the preparation may vary from batch to batch due to the natural intrinsic variability. (4) The preparation is a mixture of related constituents reflecting the natural variability during the extraction process, but the mixture may be standardized or quantified [27].

Some refined extracts can only be evaluated considering additional information such as the manufacturing process [27]. As regards the extraction solvents, ethanol and water or combinations thereof are most frequently used for an herbal preparation. Ethanol of chemical origin may contain benzene as an impurity whereas ethanol obtained from fermentation does not normally contain any benzene. Therefore, the production process of ethanol used in herbal preparations should be taken into account when considering the need for tests in the specification to control the benzene content and the frequency of testing due to benzene classified as class I solvent in the Ph. Eur. Ethanol complying with the Ph. Eur. should contain no more than 2 ppm of benzene, which is the limit requirement of the International Council for Harmonisation of Technical Requirements for Pharmaceuticals for Human Use (ICH). The content of benzene in solvents used in the manufacture of herbal preparations, which are not covered by monographs in the Ph. Eur., should, preferably, not exceed the ICH limit (2 ppm). Where solvents exceeding the ICH limit are used, potential benzene residues should be identified and quantified [27].

### Comparison of the quality requirements for Chinese and European herbal medicinal products

The ChP contains many monographs of Chinese herbal medicinal products, however, the Ph. Eur. does not have any monograph of herbal medicinal products, except requirements or directions for the dosage forms such as those in tablets and capsules, which applies to the products in the given class. The rest, such as quality of herbal medicinal products, specifications: test procedures and acceptance criteria for herbal medicinal products, are established by the EMA and defined in the scientific guideline of the EMA, which is essential to the finished products for quality control in a manufacturing chain of herbal medicinal products. During the development of herbal medicinal products, some quality-related questions could be raised, which could be answered by the EMA. Therefore, the organization of scientific advice with the EMA or the EU national competent authorities is a very useful route for pharmaceutical industries, whilst it is a pity that Chinese pharmaceutical industries seldom use this opportunity.

**Fig. 2:**
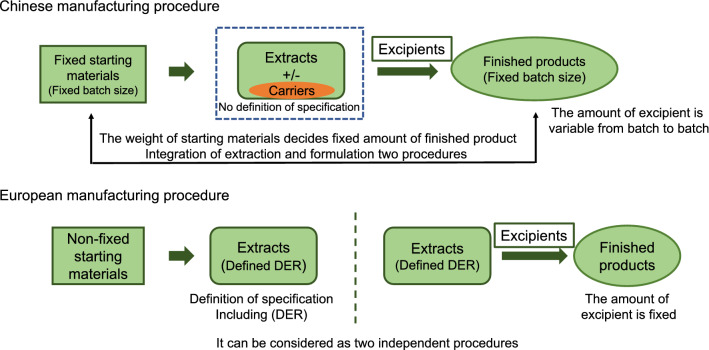
Different concepts for manufacturing process of herbal medicinal products between the EU and China. The largest difference is that the manufacturing process from herbal drugs to finished products can be considered as one procedure in China and two relatively independent procedures in the EU, which can lead to the variation in composition of the finished products

With the manufacturing process of herbal medicinal products, general principles for herbal preparation e.g., water or ethanol extract, and dosage forms e.g., tablet or capsule, are rather similar in the EMA and the ChP. However, there is also a significant difference between them, which often causes re-development (also named the second development of manufacturing process) for Chinese herbal medicinal products when applying for registration of herbal medicinal products or food supplements in the EU. For production of an herbal medicinal product, there are normally two stages, one is for the manufacture of extracts, namely intermediate, and the other is for finished products, such as table, capsule and liquids. In the EMA, the two stages are relatively independent, and herbal extracts are required to be defined with full specifications and stability tests (Fig. 2). And the compositions, namely the amounts of herbal extracts and various excipients are fixed for each finished product. But in the ChP, different situations could be found that the two stages are normally in one continuous workflow, which integrates the two stages into one entire manufacturing process from herbal substances to finished products. In this case, when preparing a technical dossier for manufacturing for the EU, the “herbal extracts” are artificially defined with specifications and stability tests. This can cause a problem, namely the composition of the individual finished medicinal product is not fixed. It is well reported that environmental growth conditions can significantly influence the composition of herbal materials [28–31]. Due to this nature of herbal drugs variation, in the Chinese pharmaceutical industrial extraction processes there is a frequent variation in extraction rate, thus the addition of excipients is used for the compensation of this variation (Fig. 2). This is a big hurdle for application for registration of Chinese herbal medicinal products in the EU when the original Chinese pharmaceutical manufacture procedure is directly used in technique dossier. This is because according to the basic requirement for registration of a medicinal product in the EU, the composition of an herbal medicinal product should be relatively unchanged or with very narrow variations from batch to batch and during the storage.

For herbal medicinal products that contain constituents of known therapeutic activity, the variation should not exceed ± 5% of the declared assay value at release and at the end of shelf-life. For those where the constituents of their therapeutic activity are unknown, the variation should not surpass ± 10% of the declared assay value at release and at the end of shelf-life. However, broader limits could be acceptable if justified, which is a case-by-case decision. For the majority of multi-herbal combinations of Chinese herbal medicinal products, simultaneous extraction of several herbal substances with the same extraction solvent such as water or ethanol/water mixture is a normal manufacturing procedure. For quality control, these mixed extracts should fulfil the same requirements as single herbal extracts and each of the individual extracts within the mixture should be qualitatively and quantitatively analyzed in principle.

Quality of herbal medicinal products is closely related to consumers’ benefits. Especially for herbal medicinal products containing toxic constituents, more attention needs to be paid to their quality control. Pyrrolizidine alkaloids (PAs) are nitrogen-containing compounds that occur naturally in plants. The group of PAs with 1,2-unsaturated structural feature are known to have hepatotoxicity, genotoxicity, carcinogenic potential, etc. In 2016, the EMA released an official statement on contamination of herbal medicinal products/traditional herbal medicinal products with PAs. Patient exposure to PAs from medicinal products should be as low as possible and must not exceed the maximum daily intake agreed by the competent authority, which is 1.0 μg or 0.35 μg according to the EMA/HMPC/328782/2016 [32]. In 2021, the monograph of contaminant PAs (2.8.26) comes into force in the Ph. Eur., in which the analytical method is introduced (liquid chromatography-tandem mass spectrometry). Now, the herbal medicinal products in the EU need to be tested for PAs, and 28 of them are required to be detected and quantified (Additional file 1: Table S5). It was reported [33] that some Chinese herbal drugs such as Senecionis scandentis hebra, Eupatorii herba, Arnebiae radix, and Farfarae flos also contain this kind of constituents. In the ChP, adonifoline (C_18_H_23_NO_7_), a PA, in Senecionis scandentis hebra is required to be not more than 0.004% in dried herbal drug.

Another group of toxic components is known as aristolochic acids isolated from plants of *Aristolochia* genus (family Aristolochiaceae) with severely nephrotoxic, mutagenic, and carcinogenic effects. Aristolochic acids include 3, 4-methylenedioxy-8-methoxy-10-nitrophenanthrene-1-carboxylic acid as aristolochic acid I, and its demethoxylated derivative as aristolochic acid II. The aristolochic acids appear to occur throughout the plants and have been found in the root, stem, herb and fruit. Herbal drugs containing aristolochic acids are forbidden from use in herbal medicinal products in the EU, except some of the EU member states that permit homoeopathic products containing *Aristolochia* species. However, it should be used with high dilutions so that it is considered to be without health hazard [34]. The test method for aristolochic acids in herbal drugs is in a separated monograph in the Ph. Eur., which can be used for any herbal drug suspected to have the contamination of aristolochic acids. This contamination can be caused either by the herbal drug itself or its growth environments. In the ChP, Chinese herbal drugs containing aristolochic acids such as Aristolochiae manshuriensis caulis, Aristolochiae fangchi radix, Aristolochiae radix, Aristolochiae fructus and Aristolochiae herba have been removed gradually, but Asari radix et rhizoma, an herbal drug from *Aristolochia* genus, is retained because both its root and rhizome hardly contain aristolochic acids. Meanwhile, any Chinese herbal medicinal products containing *Aristolochia* species for oral administration are kept under control as prescription drugs in China.

In summary, quality requirements of herbal drug in China and the EU are similar. Their required items are generally the same, but the specific markers and limits may be different. However, the technical terms to define and express the extraction rate are different for the preparation of herbal drug extracts, which leads to the differences in the manufacturing process of herbal medicinal products. Misunderstanding this may cause the failure when applying for registration of CHMs in the EU, which should be noted by related Chinese enterprises.

## Regulatory pathways for herbal medicinal products to market in the EU

### Registration for herbal medicinal products in the EU

There are three main regulatory pathways for bringing an herbal medicinal product to market in the EU. They are traditional use registration, well-established use MA, and stand-alone or mixed application, respectively [35].

#### Traditional use registration

Traditional use registration, also called simplified registration procedure, was introduced by Directive 2004/24/EC. This registration is designed for herbal medicinal products with a long tradition of medicinal use (at least 30 years, including 15 years in the EU), which can be used without the supervision of a medical practitioner and are not administered by injection. For registration of a Chinese herbal medicinal product with the simplified registration procedure, supporting evidence of traditional use in the EU is always an unavoidable challenge, which must be demonstrated with bibliographic or expert evidence, for example:

- marketing data, brochures, etc.;
- references to manuals, such as the Farmacotherapeutisch compendium (J. van Hellemont), Pharmacotherapeutisch vademecum, old editions by Martindale, Rote Liste, Informatorium;
- official lists of licensed/authorised traditional herbal medicinal products in other member states of the EU;
- reports from experts such as pharmacognosists, herbalists, doctors and pharmacists;
- national pharmacopoeias of member states that were used in the past, or the British Herbal Pharmacopoeia;
- Hoppe HA. Drogenkunde;
- List PH, Hörhammer L. Hagers Handbuch der Pharmaceutischen Praxis. Für Apotheker Arzneimittelhersteller Ärzte und Medizinalbeamte;
- Madaus G. Lehrbuch der biologischen Heilmittel

#### Well-established use marketing authorization (MA)

Well-established use is applicable to a medicinal product having published scientific literature or studied data with an acceptable level of efficacy and safety, as well as having at least 10 years of medicinal use history in the EU. For this category, it is very likely that most of CMHs are not suitable.

#### Stand-alone full MA or mixed application for MA

Stand-alone or mixed application is only applicable for novel herbal medicinal products based on own research and development. This application requires a complete dossier of clinical data, and it is not different from new western medicine MA application. There is also an option for combination with bibliographic data.

Traditional use registration can be applied in national competent authority of a Member State for national, mutual recognition or decentralized procedures. Well-established use MA and stand-alone or mixed application for MA can be applied not only in national, mutual recognition and decentralized procedures, but also centralized procedure in the EMA. With the national procedure, the traditional use registration can be applied for competent authority of a Member State and is only valid in this country. For the mutual recognition procedure, the registration application is based on the recognition of a pre-existing national registration by the Reference Member State (RMS), and then submitted to another or more EU countries. For the decentralized procedure, the registration application is submitted simultaneously in several EU countries, one being chosen as RMS.

To bring a Chinese herbal medicinal product to the EU medicinal market, traditional use registration is much more significant than the other two regulatory pathways. So far, there have been 7 Chinese herbal medicinal products successfully registered by simplified registration procedure (Directive 2004 24/EC) in the EU, including 5 single herbal medicinal products and 2 multi-herbal combination products (Table 2). The manufacturing and quality of herbal medicinal products for traditional use registration have the same requirements as applications for MA. For indications, the traditional use registration is strict in the field of over-the-counter (OTC) and self-medication area compared with well-established use MA and stand-alone full MA or mixed application for MA (Table 3).

**Table 2:** Traditional use registration of Chinese herbal medicinal products in the EU

Chinese herbal medicinal products	Active ingredients	Indications	Companies	Place for registration
Diao Xinxue Kang Capsules	Dioscoreae nipponicae rhizoma	Relief of headache and muscle pain and cramps in neck, back and legs	Chengdu Di'ao Pharmaceutical Group Co., Ltd, China	The Netherlands
Phynova Joint and Muscle Pain Relief Tablets	Sigesbeckiae orientalis herba	Relief of backache, minor sports injuries, rheumatic or muscular pains, and general aches and pains in the muscle and joints	Phynova Group Ltd, United Kingdom	United Kingdom
Danshen Capsules	Salviae milthiorrhizae radix et rhizoma	Relief of mild menstrual pains	Tianjin Tasly Pharmaceutical Co., Ltd, China	The Netherlands
Phynova Cold and Flu Relief Powder for Oral Solution	Isatidis radix	Relief of cold and flu	Guangzhou Xiangxue Pharmaceutical Co., Ltd, China	United Kingdom
Yufeng Ningxin Tablets	Pueraria lobata radix	Relief of headache and neck and shoulder muscle pain	Beijing TongRenTang Co. Ltd, China	The Netherlands
Yamato Gast Film-coated Tablets	Ginseng radix, Atractylodis rhizoma, Poria, Pinelliae rhizoma, Citri unshiu pericarpium, Jujubae fructus, Liquiritiae radix, and Zingiberis rhizoma	Relief of mild gastrointestinal disorders such as loss of appetite, malaise, bloating and bloating	Ominedo Pharmaceutical Industry Co., Ltd, Japan	Germany
Xiao Yao coated Tablets	Bupleuri radix, Paeoniae radix alba, Angelicae sinensis radix, Atractylodis macrocephalae rhizoma, and Liquiritiae radix	Relief of mental stress and exhaustion, such as low mood and loss of appetite	Tianjin Tasly Pharmaceutical Co., Ltd, China	The Netherlands

**Table 3:** Marketing authorization *vs.* simplified registration for herbal medicinal products in the EU

Categories	Market authorization for herbal medicinal products (2001/83/EC)	Simplified registration for herbal medicinal products (2004/24 /EC)
Indications	No specific limitations	Limited to self-medication and OTC
Traditional use evidence	No requirement for stand-alone or mixed application At least 10 years (medicinal use) for well-established use	30 years outside the EU including 15 years in the EU
Dosage form	No specific limitations	Limited to oral, external use and inhalation
Dossier requirements	Pre-clinical (new tests) and clinical data (new trials), or combined with some bibliographic data, Pharmacovigilance	Bibliographic documents, or safety tests, Pharmacovigilance
Quality control	GACP, GMP and CMC	GACP, GMP and CMC

*OTC* Over-the-Counter, *GACP* Good Agricultural and Collection Practice, *GMP* Good Manufacturing Practice, *CMC* Chemistry Manufacturing and Controls

### Legal status of TCM granules in the EU

TCM granules are granules manufactured based on single Chinese herbal drug via extraction with heating water, separation, concentration, dry, and granulation. They are used in the clinic, under the guidance of the theory of TCM. TCM granules possess the basic properties of decoction and conform to the general rules of granules, which are used in accordance with Chinese herbal drugs as clinical formula.

In most EU member states, TCM granules are regarded as food supplements. However, in Germany TCM granules are considered as medicine [36], this type of product should be regulated as medicinal product from different aspects including production, quality control and distribution channel etc. This difference in regulation and management in different member states is not yet harmonized. Generally speaking, the high product quality management system in Germany is respected. In 2016, the German Federal Administrative Court made a decision on the legal status of TCM granules that TCM granules are judged to be “Präsentationsarzneimittel”. Präsentationsarzneimittel (German) is translated as “presentation medicinal products” in English, which means that TCM granules are medicinal products that cannot be directly used by consumers but require a doctor or professional practitioner’s prescriptions. Therefore, their medicinal use is based on their designation or presentation. For quality control, quality monographs of TCM granules are in the process of establishment by the Deutscher Arzneimittel-Codex (DAC)/German Drug Codex. The DAC has been published by the Federal Union of German Associations of Pharmacists since 1967 and is a supplement book to the German Pharmacopoeia. Thus, the DAC closes the gap in the quality and is an important source of auditing standards for drug manufacturers. It also contains numerous monographs of medicinal drugs. In order to import TCM granules to the German market, an import permission and European Good Manufacturing Practice inspection are required, even if they are only given to pharmacies and not directly to the end consumer. Currently the TCM granules in the German market are imported via the third country, not directly from China. Thus, now would be the right time to do so, as there is a huge opportunity for Chinese pharmaceutical industries that produce TCM granules to explore their market potential in the EU.

## Conclusions and perspectives

The quality requirements of CHMs play a critical role in the internationalization of TCM. In particular, the pharmacopoeias of some countries have established basic requirements for the quality control of CHMs, which have become the threshold for the market access of CHMs in these countries. Comparing the quality standards of CHMs in different pharmacopoeias, it is obvious that they are different in terms of the species and quantities of CHMs as well as the items stipulated in monographs. Moreover, the numbers and requirements of CHMs recorded in pharmacopoeias of other countries are significantly different from those in the ChP. This status quo has greatly restricted the acceptance of TCM at international level.

Compared with western medicine, CHM has its unique features. It needs to be used compatibly under the guidance of the theoretical system of TCM. This should take into consideration the quality control of CHM preparations. However, due to the lack of evidence for effective substance in many CHMs and the limitations of existing technologies, there is still no set of matching standards available. In existing quality standards of CHMs, quality marker usually is just one or two components or a class of substances, such as volatile oil, which is often impossible to accurately assess the quality of CHMs. Therefore, how to conduct systematic research on the quality markers of CHMs and achieve technological breakthroughs, so as to establish a set of leading quality control technologies and international standards, will become a research hotspot and challenge in the field of quality control of CHMs. Realization of this goal will greatly promote the internationalization of TCM and benefit human health. It is worth stating that systems biology and network pharmacology, which focuses on complex interactions in biological systems and studies drug actions and interactions with multiple targets, have the potential to be useful and important tools for systematic research of effective substances of CHMs [37, 38]. Above all, evidence based clinical investigation is currently considered to be a golden standard for scientific acceptance of CHMs [39]. From this perspective, how to bridge western medical science with TCM theory and philosophy is still considered to be a challenge. Chinese medicine’s prevention and treatment strategy is multi-target and non-linear approach. Research on the combination of different disciplines with systems pharmacology may shed some light for future medicinal sciences.

**Fig. 3:**
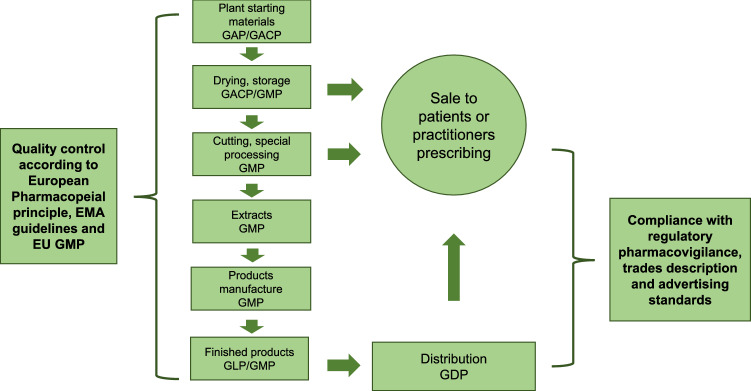
Key steps in monitoring the quality of herbal medicinal products. From herbal drugs to finished products, there is a continuous process for quality control at each step from manufacturing to marketing. Collectively, these quality controls are the critical steps that guarantee the quality of finished products. GAP/GACP: Good agricultural/collecting practice; GLP: Good laboratory practice; GMP: Good manufacturing practice; GDP: Good distribution practice

The quality control of CHMs is required in all aspects of the production of Chinese herbal medicinal products (Fig. 3). The establishment of the EU quality standards and official registration for CHMs will be the first step into the EU market. Firstly, the quality of CHMs should be controlled according to the Ph. Eur. principles and the EMA guidelines. The quality of Chinese herbal drugs and extracts in the EU is mainly defined in the Ph. Eur., while the efficacy and safety guidelines of herbal medicinal products are established by the EMA. In the EU, there are three main regulatory pathways for bringing an herbal medicinal product to market, including traditional use registration, well-established use MA, and stand-alone full MA or mixed application for MA. They are applicable according to specific situations of CHMs, such as medicinal use history and method of administration. It is important to note that the different registration pathways can be applied in different procedures which refer to national, mutual recognition, decentralized procedures and centralized procedure in the EMA. As a matter of fact, there have always been some obstacles to CHMs entering the European market. This is largely due to the unclear understandings of quality control of CHMs in the EU by Chinese pharmaceutical companies. Therefore, in this review, the differences have been elaborated in detail by comparing the quality standards of CHMs in the EU and China. Chinese enterprises should focus on and have a good grasp of these differences to save costs and to enable a smoother operation/transition when introducing their Chinese herbal medicinal products into the EU market. In addition, TCM granules, as a special case, are elaborated separately in this review. In the EU, TCM granules are regarded as food supplements, except in Germany, where TCM granules are managed and monitored as medicinal products, which needs to get the attention of related Chinese enterprises.

## Supplementary Information

**Additional file 1: Table S1.** The limit standards of 69 pesticides in the Ph. Eur. (10th edition)**. Table S2.** The limit standardcs of 33 pesticides in the ChP (2020 edition). **Table S3.** The Chinese herbal drugs required test of heavy metals in the ChP (2020 edition). **Table S4.** The Chinese herbal drugs required test of aflatoxins in the ChP (2020 edition). **Table S5.** Target PAs required to be tested in herbal medicinal products.

## Data Availability

Not applicable.

## Abbreviations

CHMs: Chinese herbal medicines
ChP: Chinese Pharmacopoeia
CMC: Chemistry manufacturing and controls
DAC: Deutscher Arzneimittel-Codex
DER: Drug extract ratio
DER _genuine_: Drug extract ratio _genuine_
EDQM: European Directorate for the Quality of Medicines and Health Care
EMA: European Medicines Agency
EU: European Union
GACP: Good agricultural and collection practice
GDP: Good distribution practice
GLP: Good laboratory practice
GMP: Good manufacturing practice
ICH: International Council for Harmonisation of Technical Requirements for Pharmaceuticals for Human Use
JP: Japanese Pharmacopoeia
MA: Marketing authorization
OTC: Over-the-counter
PAs: Pyrrolizidine alkaloids
Ph. Eur.: European Pharmacopoeia
RMS: Reference Member State
TCM: Traditional Chinese medicine
TLC: Thin layer chromatography
USP-NF: United States Pharmacopeia-National Formula
