# Supplementary material for: Key quality factors for Chinese herbal medicines entering the EU market
Source: Chin Med. 2022 Feb 22;17:29. doi: 10.1186/s13020-022-00583-x (PMC8861989; doi:10.1186/s13020-022-00583-x)
Supplement: Supplementary file 1 — Additional file 1: Table S1. The limit standards of 69 pesticides in the Ph. Eur. (10th edition). Table S2. The limit standardcs of 33 pesticides in the ChP (2020 edition). Table S3. The Chinese herbal drugs required test of heavy metals in the ChP (2020 edition). Table S4. The Chinese herbal drugs required test of aflatoxins in the ChP (2020 edition). Table S5. Target PAs required to be tested in herbal medicinal products. [file 13020_2022_583_MOESM1_ESM.docx]

**Supplement information for manuscript:**

**Key quality factors for** **Chinese herbal medicines entering the EU market**

Mei Wang^1^^,3#^*, Pei-Fen Yao^2#^, Peng-Yue Sun^3^, Wen Liang^3^, Xiao-Jia Chen^2^*

*^1^* *LU-European Center for Chinese Medicine and Natural Compounds, Institute of Biology,* *Leiden University, Sylviusweg72, 2333BE, Leiden, The Netherlands*

*^2^ Institute of Chinese Medical Sciences, and State Key Laboratory of Quality Research in Chinese Medicine, University of Macau, Macao SAR, China*

*^3^ SU Biomedicine,* *BioPartner Center 3, Galileiweg 8, Leiden Bio Science Park, 2333 BD Leiden, the Netherlands.*

^#^ The authors contributed equally to this work.

***Correspondence**

**Dr. Mei Wang**

LU-European Center for Chinese Medicine and Natural Compounds, Institute of Biology, Leiden University, Sylviusweg72, 2333BE, Leiden, The Netherlands

Tel: +31 (0) 715275027; E-mail: [Mei.Wang@Subiomedicine.com](mailto:Mei.Wang@Subiomedicine.com)

**Dr. Xiao-Jia Chen**

Institute of Chinese Medical Sciences, and State Key Laboratory of Quality Research in Chinese Medicine, University of Macau, Avenida da Universidade, Taipa, Macao SAR, PR China

Tel: +853-8822 4915; E-mail: [XiaojiaChen@um.edu.mo](mailto:xiaojiachen@um.edu.mo)

**Table S1. The limit standards of 69 pesticides in the Ph. Eur. (10th edition)**

| No. | Substance | Limit (mg/kg) |
| --- | --- | --- |
| 1 | Acephate | 0.1 |
| 2 | Alachlor | 0.05 |
| 3 | Aldrin and dieldrin (sum of) | 0.05 |
| 4 | Azinphos-ethyl | 0.1 |
| 5 | Arinphos-methyl | 1 |
| 6 | Bromophos-ethyl | 0.05 |
| 7 | Bromophos-methyl | 0.05 |
| 8 | Brompropylate | 3 |
| 9 | Chlordane (sum of *cis*-, *trans*- and oxychlordane) | 0.05 |
| 10 | Chlorfenvinphos | 0.5 |
| 11 | Chlorpyriphos-ethyl | 0.2 |
| 12 | Chlorpyriphos-methyl | 0.1 |
| 13 | Chlorthal-dimethyl | 0.01 |
| 14 | Cyfluthrin (sum of) | 0.1 |
| 15 | λ-Cyhalothrin | 1 |
| 16 | Cypermethrin and isomers (sum of) | 1 |
| 17 | DDT (sum of *o,p'*-DDE, *p,p'*-DDE, *o,p'*-DDT, *p,p'*-DDT, *o,p'-*TDE and *p,p'*-TDE) | 1 |
| 18 | Deltamethrin | 0.5 |
| 19 | Diazinon | 0.5 |
| 20 | Dichlofluanid | 0.1 |
| 21 | Dichlorvos | 1 |
| 22 | Dicofol | 0.5 |
| 23 | Dimethoate and omethoate (sum of) | 0.1 |
| 24 | Dithiocarbamates (expressed as CS_2_) | 2 |
| 25 | Endosulfan (sum of isomers and endosulfan sulfate) | 3 |
| 26 | Endrin | 0.05 |
| 27 | Ethion | 2 |
| 28 | Etrimphos | 0.05 |
| 29 | Fenchlorophos (sum of fenchlorophos and fenchlorophos-oxon) | 0.1 |
| 30 | Fenitrothion | 0.5 |
| 31 | Fenpropathrin | 0.03 |
| 32 | Fensulfothion (sum of fensulfothion, fensulfothion-oxon, fensulfothion-oxonsulfon and fensulfothion-sulfon) | 0.05 |
| 33 | Fenthion (sum of fenthion, fenthion-oxon, fenthion-oxon-sulfon, fenthion-oxon-sulfoxid, fenthion-sulfon and fenthion-sulfoxid) | 0.05 |
| 34 | Fenvalerate | 1.5 |
| 35 | Flucytrinate | 0.05 |
| 36 | τ-Fluvalinate | 0.05 |
| 37 | Fonophos | 0.05 |
| 38 | Heptachlor (sum of heptachlor, *cis*-heptachlorepoxide and *trans*-heptachlorepoxide) | 0.05 |
| 39 | Hexachlorbenzene | 0.1 |
| 40 | Hexachlorocyclohexane (sum of isomers α-, β-, δ- and ε) | 0.3 |
| 41 | Lindan (γ-hexachlorocyclohexane) | 0.6 |
| 42 | Malathion and malaoxon (sum of) | 1 |
| 43 | Mecarbam | 0.05 |
| 44 | Methacriphos | 0.05 |
| 45 | Methamidophos | 0.05 |
| 46 | Methidathion | 0.2 |
| 47 | Methoxychlor | 0.05 |
| 48 | Mirex | 0.01 |
| 49 | Monocrotophos | 0.1 |
| 50 | Parathion-ethyl and paraoxon-ethyl (sum of) | 0.5 |
| 51 | Parathion-methyl and paraoxon-methyl (sum of) | 0.2 |
| 52 | Pendimethalin | 0.5 |
| 53 | Pentachloranisol | 0.01 |
| 54 | Permethrin and isomers (sum of) | 1 |
| 55 | Phosalone | 0.1 |
| 56 | Phosmet | 0.05 |
| 57 | Piperonyl butoxide | 3 |
| 58 | Pirimiphos-ethyl | 0.05 |
| 59 | Pirimiphos-methyl (sum of pirimiphos-methyl and *N*-desethyl-pirimiphos-methyl) | 4 |
| 60 | Procymidone | 0.1 |
| 61 | Profenophos | 0.1 |
| 62 | Prothiophos | 0.05 |
| 63 | Pyrethrum (sum of cinerin I, cinerin II, jasmolin I, Jasmolin II, pyrethrin I and pyrethrin II) | 3 |
| 64 | Quinalphos | 0.05 |
| 65 | Quintozene (sum of quintozene, pentachloraniline and methyl penthachlorphenyl sulfide) | 1 |
| 66 | S-421 | 0.02 |
| 67 | Tecnazene | 0.05 |
| 68 | Tetradifon | 0.3 |
| 69 | Vinclozolin | 0.4 |

**Table S2. The limit standards of 33 pesticides in the ChP (2020 edition)**

| No. | Substance | Limit (mg/kg) |
| --- | --- | --- |
| 1 | Aldicarb (Sum of aldicarb, aldicarb-sulfone and aldicarb-sulfoxide, expressed as aldicarb) | 0.1 |
| 2 | Aldrin | 0.05 |
| 3 | BHC (sum of *α*-BHC, *β*-BHC, *γ*-BHC and *δ*-BHC, expressed as BHC) | 0.1 |
| 4 | Cadusafos | 0.02 |
| 5 | Carbofuran (sum of carbofuran and carbofuran-3-hydroxy, expressed as carbofuran) | 0.05 |
| 6 | Chlordimeform | 0.02 |
| 7 | Chlorsulfuron | 0.05 |
| 8 | Coumaphos | 0.05 |
| 9 | DDT (sum of *p,p'*-DDT, *o,p'*-DDT, *p,p'*- DDE and *p,p'*-DDD, expressed as DDT) | 0.1 |
| 10 | Demeton (sum of *O*-demeton and *S*-demeton) | 0.02 |
| 11 | Dicofol (sum of *o,p'*-dicofol and *p,p'*-dicofol, expressed as dicofol) | 0.2 |
| 12 | Dieldrin | 0.05 |
| 13 | Endosulfan (sum of *α*-endosulfan, *β*-endosulfan and endosulfan sulfate, expressed as endosulfan) | 0.05 |
| 14 | Ethametsulfuron-methyl | 0.05 |
| 15 | Ethoprophos | 0.02 |
| 16 | Fenamiphos (Sum of fenamiphos, fenamiphos-sulfoxide and fenamiphos-sulphone, expressed as fenamiphos) | 0.02 |
| 17 | Fipronil (sum of fipronil, fipronil-desulfinyl, fipronil-sulfone and fipronil-sulfoxide, expressed as fipronil) | 0.02 |
| 18 | Fonofos | 0.02 |
| 19 | Isazofos | 0.01 |
| 20 | Isocarbophos | 0.05 |
| 21 | Isofenphos-methyl | 0.02 |
| 22 | Methamidophos | 0.05 |
| 23 | Metsulfuron-methyl | 0.05 |
| 24 | Monocrotophos | 0.03 |
| 25 | Nitrofen | 0.05 |
| 26 | Parathion-ethyl | 0.02 |
| 27 | Parathion-methyl | 0.02 |
| 28 | Phorate (sum of phorate, phorate-sulfone and phorate-sulfoxide, expressed as phorate) | 0.02 |
| 29 | Phosfolan | 0.03 |
| 30 | Phosfolan-methyl | 0.03 |
| 31 | Phosphamidon | 0.05 |
| 32 | Sulfotep | 0.02 |
| 33 | Terbufos (sum of terbufos, terbufos-sulfone and terbufos-sulfoxide, expressed as terbufos) | 0.02 |

**Table S3. The Chinese herbal drugs required test of heavy metals in the ChP (2020 edition)**

| No. | Latin Name | Pinyin Name |
| --- | --- | --- |
| 1 | Angelicae dahuricae radix | Baizhi |
| 2 | Angelicae sinensis radix | Danggui |
| 3 | Astragali radix | Huangqi |
| 4 | Corni fructus | Shanzhuyu |
| 5 | Crataegi fructus | Shanzha |
| 6 | Gardeniae fructus | Zhizi |
| 7 | Ginseng radix et rhizoma | Renshen |
| 8 | Glycyrrhizae radix et rhizoma | Gancao |
| 9 | Laminariae thallus or Eckloniae thallus | Kunbu |
| 10 | Lonicerae japonicae flos | Jinyinhua |
| 11 | Lycii fructus | Gouqizi |
| 12 | Notoginseng radix et rhizoma | Sanqi |
| 13 | Paeoniae radix alba | Baishao |
| 14 | Panacis quinquefolii radix | Xiyangshen |
| 15 | Persicae semen | Taoren |
| 16 | Polygonati rhizoma | Huangjing |
| 17 | Puerariae lobatae radix | Gegen |
| 18 | Salviae miltiorrhizae radix et rhizoma | Danshen |
| 19 | Sargassum | Haizao |
| 20 | Ziziphi spinosae semen | Suanzaoren |

**Table S4. The Chinese herbal drugs required test of aflatoxins in the ChP (2020 edition)**

| No. | Latin Name | Pinyin Name |
| --- | --- | --- |
| 1 | Arecae semen | Binglang |
| 2 | Cassiae semen | Juemingzi |
| 3 | Citri reticulatae pericarpium | Chenpi |
| 4 | Coicis semen | Yiyiren |
| 5 | Corydalis rhizoma | Yanhusuo |
| 6 | Hordei fructus germinatus | Maiya |
| 7 | Jujubae fructus | Dazao |
| 8 | Myristicae semen | Roudoukou |
| 9 | Nelumbinis semen | Lianzi |
| 10 | Persicae semen | Taoren |
| 11 | Platycladi semen | Baiziren |
| 12 | Polygalae radix | Yuanzhi |
| 13 | Quisqualis fructus | Shijunzi |
| 14 | Sterculiae lychnophorae semen | Pangdahai |
| 15 | Strychni semen | Maqianzi |
| 16 | Ziziphi spinosae semen | Suanzaoren |

**Table S5. Target PAs required to be tested in herbal medicinal products**

| No. | Target PAs |
| --- | --- |
| 1 | Echimidine |
| 2 | Echimidine-*N*-oxide |
| 3 | Erucifoline |
| 4 | Erucifoline-*N*-oxide |
| 5 | Europine |
| 6 | Europine-*N*-oxide |
| 7 | Heliotrine |
| 8 | Heliotrine-*N*-oxide |
| 9 | Intermedine |
| 10 | Intermedine-*N*-oxide |
| 11 | Jacobine |
| 12 | Jacobine-*N*-oxide |
| 13 | Lasiocarpine |
| 14 | Lasiocarpine-*N*-oxide |
| 15 | Lycopsamine |
| 16 | Lycopsamine-*N*-oxide |
| 17 | Monocrotaline |
| 18 | Monocrotaline-*N*-oxide |
| 19 | Retrorsine |
| 20 | Retrorsine-*N*-oxide |
| 21 | Senecionine |
| 22 | Senecionine-*N*-oxide |
| 23 | Seneciphylline |
| 24 | Seneciphylline-*N*-oxide |
| 25 | Senecivernine |
| 26 | Senecivernine-*N*-oxide |
| 27 | Senkirkine |
| 28 | Trichodesmine |
